# Measuring traveling wave velocity in the basilar membrane as a potential indicator of endolymphatic hydrops in definite Ménière’s disease: a narrative review

**DOI:** 10.3389/fneur.2024.1406617

**Published:** 2024-12-18

**Authors:** Xingqian Shen, Hui Pan, Linlin Wang, Wen Xie, Yangming Leng, Bo Liu, Hongjun Xiao

**Affiliations:** Department of Otorhinolaryngology-Head and Neck Surgery, Tongji Medical College, Union Hospital, Huazhong University of Science and Technology, Wuhan, China

**Keywords:** Ménière’s disease, cochlear hydrops analysis masking procedure, traveling wave velocity, diagnoses, endolymphatic hydrops

## Abstract

**Background:**

The pathological hallmark of Ménière’s disease is endolymphatic hydrops, which can lead to an increase in basilar membrane stiffness and, consequently, an acceleration of the traveling wave of sound. The cochlear hydrops analysis masking procedure (CHAMP), which is an auditory brainstem response test masked at various frequencies with high-pass noise masking, uses the principle of the traveling wave velocity theory to determine the presence of endolymphatic hydrops.

**Purpose:**

This study aimed to review the previous results of the CHAMP, expound the principles and key indicators, and discuss its clinical significance in diagnosing Ménière’s disease.

**Methods:**

A narrative review was performed to revisit the principles of the CHAMP test, procedures, and clinical application results in diagnosing Ménière’s disease.

**Results:**

According to the published literature, the CHAMP has a specificity of 31–100%, a sensitivity of 28–100%, and a diagnostic accuracy of 30–100% for the diagnosis of Ménière’s disease, including patients with definite, probable, or possible Ménière’s disease in various studies. These inconsistent results were due to subject inclusion criteria, variable settings, waveform identification, and other factors. Nevertheless, as an electrophysiological technique, the CHAMP may have a relatively high diagnostic value in patients with a definite Ménière’s disease.

**Conclusion:**

The CHAMP is still potentially useful for studying the pathophysiology of hydropic ear diseases since the procedure can measure the traveling wave velocity of the basilar membrane in the era of imaging to detect endolymphatic hydrops for Ménière’s disease.

## 1 Introduction

Ménière’s disease (MD) is an idiopathic inner ear condition, of which the pathological feature is endolymphatic hydrops (ELH) (1, 2), characterized by the clinical presentation of episodic vertigo attacks, fluctuating sensorineural hearing loss (SNHL), tinnitus, and aural fullness (3). At present, the etiology and pathophysiological mechanisms of MD have not been fully elucidated. Thus far, the leading international guidelines for diagnosing and treating MD have recommended using medical history information and pure tone audiometry to diagnose MD (3). Over the years, diagnosing MD has remained challenging for clinicians due to the diversity and fluctuating nature of clinical presentations. In addition to conventional pure tone audiometry, there are also many auxiliary examinations in clinical practice to facilitate the diagnosis of MD, including various neurophysiological and radiological techniques. These often include electrocochleogram (ECochG) (4), glycerol test (5), vestibular testing (dissociation of caloric test and vHIT results) (6), and gadolinium (Gd)-enhanced magnetic resonance imaging (MRI) of inner ear (7). However, so far there is no audiological test with high specificity and sensitivity for the diagnosis of MD.

In patients with MD, the basilar membrane stiffness increases with the expansion in endolymphatic space (8), which may lead to the gain of traveling wave velocity (TWV) in cochlear hydrops. Interestingly, Don et al. (9) found that the modification of TWV can be measured by comparing the auditory brainstem response (ABR) masked at different frequencies with high-pass noise masking. Based on the above-mentioned theory of TWV, the cochlear hydrops analysis masking procedure (CHAMP) was first proposed by Don et al. (10) to evaluate patients with MD, and was considered to have 100% sensitivity and 100% specificity in distinguishing active MD in individuals. Due to its high sensitivity and specificity, the CHAMP, as a new diagnostic modality, has been regarded as a potential test to help diagnose MD (7, 11, 12). However, several follow-up findings showed inconsistent results. For example, when subjects had SNHL, the validity of the CHAMP for identifying MD was doubtful (13–20). Therefore, it is necessary to review this test and its associated findings. This narrative review aims to summarize and compare the results of previous studies, expound the principles and key indicators of the CHAMP in detail, and assess the feasibility and effectivity of the diagnosis for MD.

## 2 Principle and protocol of the CHAMP

### 2.1 Principles of the CHAMP

The CHAMP is an auditory electrophysiological technique that reflects basement membrane stiffness and response characteristics by comparing the differences in ABR wave V induced by clicks with and without high-pass noise masking. When ELH occurs, the stiffness of the basement membrane increases and the response characteristics change. Therefore, the CHAMP is used to assist in the diagnosis of MD.

**Figure 1:**
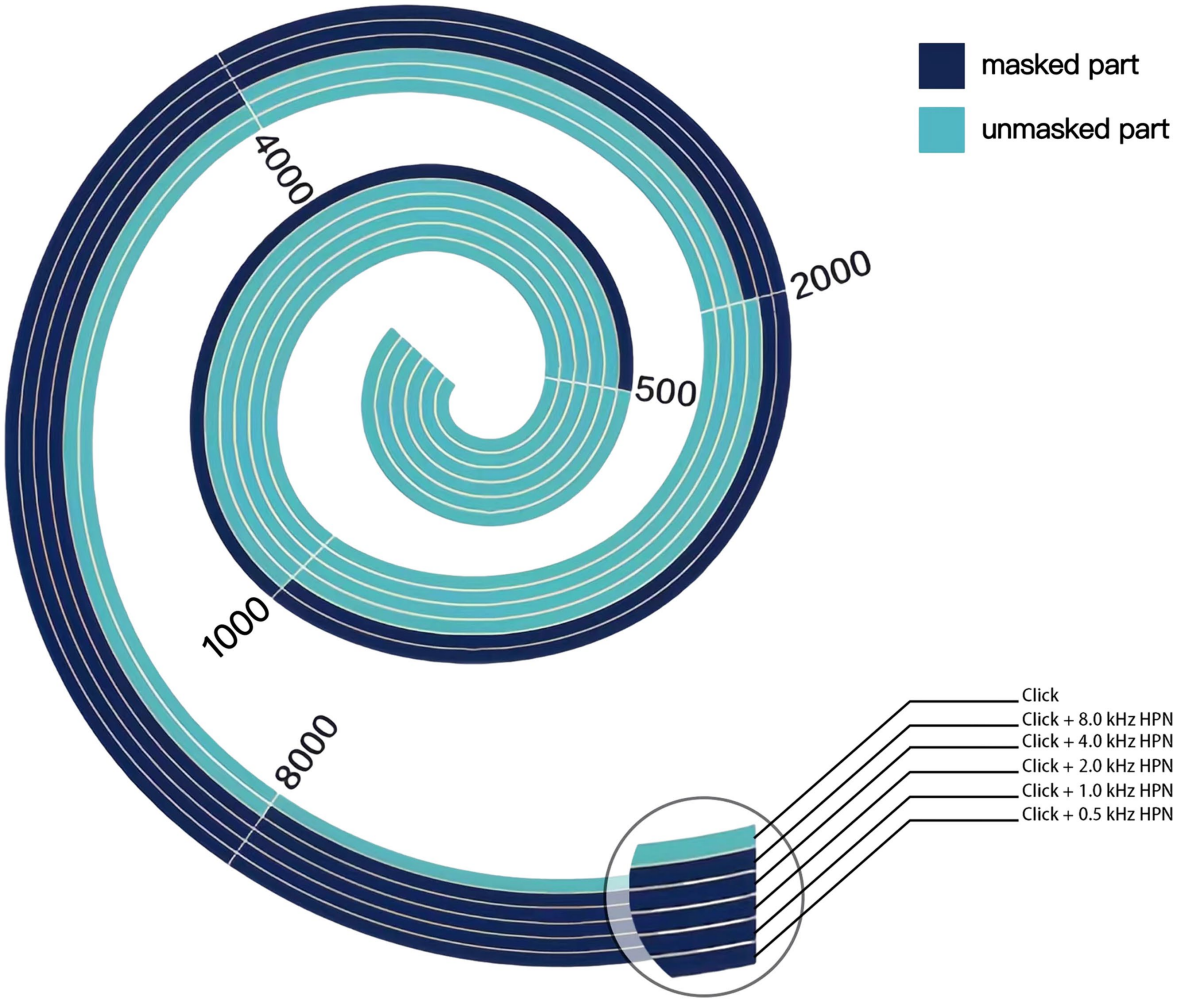
Schematic representation of basilar membrane activation under different acoustic stimulation conditions in the CHAMP. The unmasked part is in light color, while the masked part is in dark color. When a click appears alone, the entire basilar membrane is activated. In contrast, when high-pass noise is added for masking, only the unmasked part near the top is activated, from which ABR originates. As the high-pass noise cutoff gradually decreases in frequency, the unmasked part gradually decreases and moves toward the top of the cochlea. HPN, high-pass noise.

Click is a broadband acoustic signal that can activate almost the hair cells of the entire cochlea and the corresponding auditory transduction pathway, usually used as a stimulus sound to evoke ABR (21, 22). However, due to the high synchronization of neurons corresponding to the high-frequency region, the click-evoked ABR mainly comes from that, in the absence of noise masking (21). During the CHAMP test, a high-pass noise is used to mask the high-frequency components of the click, then the response comes from the unmasked part of the cochlea and the corresponding auditory pathway (10). Figure 1 schematically illustrates the region of the cochlear response to clicks under different high-pass masking noises. When the click appears alone, the hair cells of the entire cochlea are activated, while when high-pass noise is added for masking, the high-frequency region is masked, and ABR is only generated by the unmasked part of the basilar membrane and the corresponding auditory pathway. As the high-pass noise cutoff frequency decreases gradually, the cochlear response frequency gradually decreases, and the response region gradually moves to the top of the cochlea. Therefore, according to the traveling wave theory, in normal individuals, the latency of wave V under the masking of high-pass noise is prolonged in the CHAMP as the high-pass noise cutoff frequency decreases gradually because it takes longer for the sound wave to reach the top of the cochlea than the bottom of the cochlea (23).

Nevertheless, in patients with MD, the latency of wave V does not delay correspondingly as that in normal subjects (24, 25). One reason for that is the pathological feature of ELH, which causes a corresponding increase in basilar membrane stiffness gaining the TWV, thereby modifying the ABR under high-pass noise masking. Another possible explanation is that the frequency response characteristics of the cochlea are altered in MD patients, with the response area shifting to the high-frequency region of the basilar membrane due to the typical low-frequency SNHL (26). This may reduce the time it takes for low-frequency sound waves to pass through the cochlea, resulting in a shorter latency of wave V in the CHAMP test (24). Therefore, the CHAMP was initially suggested to indicate ELH, thereby facilitating the diagnosis of MD (10).

In addition, in normal subjects, compared to the click-induced ABR, the wave V amplitude is smaller in the CHAMP (13). While, in MD patients, this discrepancy of the amplitudes is not significant. For normal subjects and MD patients, first, the existence of masking in the CHAMP leads to a reduction in response nerve fibers and a weakening of electrical activity (27); second, the poor synchronization of neural activity in correlation with the low-frequency region of the cochlear also results in a lower amplitude (25). However, in MD patients, due to varying degrees of hearing loss, the amplitude of wave V induced by click alone was smaller than that of normal subjects (28, 29). For that, the difference mentioned above is smaller in MD patients.

### 2.2 Protocol of the CHAMP

In their original studies, Don et al. (10, 30) provided a detailed description of the CHAMP test. For the diagnosis of MD, they established the criterion for latency delay as (wave V latency under 0.5 kHz high-pass noise masking minus click-induced wave V latency) < 0.3 ms (10). In a subsequent study, Don et al. (30) introduced another parameter, the complex amplitude ratio (CAR), defined as [(click-induced wave V amplitude minus wave V amplitude under 0.5 kHz high-pass noise masking)/click-induced wave V amplitude] < 0.95. Both the latency delay and CAR are considered primary indicators in the CHAMP test to discriminate a group of MD patients from a group of non-MD subjects.

#### 2.2.1 Stimuli

The CHAMP is performed at six stimulus conditions: clicks presented alone (unmasked condition) and clicks presented with ipsilateral pink noise high-pass filtered at 8, 4, 2, 1, and 0.5 kHz. The click, of which polarity is the sparse wave with an intensity of 82 dB peSPL, equivalent to 60 dB nHL, is produced by applying a rectangular voltage pulse of 100 μs to the ER-2 insert earphone. The stimulus intensity of the pink noise was sufficient to mask the required intensity of 82 dB peSPL click-induced ABR (RMS level was 81 dB SPL determined by a test of a group of 10 normal hearing subjects). The pink noise is filtered to high-pass pink noise using a high-pass filter at the above cutoff points.

#### 2.2.2 CHAMP recordings

The CHAMP test recording method is similar to ABR, which was recorded differentially between the electrodes applied to the vertex and the ipsilateral mastoid. The electrode on the contralateral mastoid was used as a ground. The scalp activity was sampled for 15 ms after stimulus onset, filtered with a passband of 0.1–3 kHz, and the signal was amplified by 0.5 million times. The recording was stopped when the residual background noise was <20 nV RMS, so the variation in averaged physiological background noise could be reduced to obtain reliable neural activity. The postauricular muscle artifacts found could impact the waveform recording of CHAMP tests in the study of Shang et al. (17). The artifacts could be reduced by telling patients to relax, close their eyes, relax their jaws, and avoid placing electrodes on more muscles, but there were still noticeable postauricular muscle artifacts in some of the subjects (17).

## 3 CHAMP for diagnosis of MD

Up to the present, several investigations have documented the diagnostic utility of the CHAMP in the context of MD, revealing variations in the obtained outcomes (Table 1). According to the published literature, for the diagnosis of MD, the CHAMP has a specificity of 31–100%, a sensitivity of 28–100%, and a diagnostic accuracy of 30–100%. After examining these bibliographies, we found discrepancies in various aspects, which may be attributed to the inconsistency of the findings. Herein, we aim to specify the homogeneity and heterogeneity in these studies.

**Table 1:** Diagnostic value of CHAMP in the published literature.

Studies	Cases (MD/Control)	MD group	Diagnosis by criteria of AAO-HNS (Yes/No)	Control group	Variables settings	Excluding the non-interpretable results (Yes/No)	Sensitivity	Specificity	Diagnostic accuracy
Don et al., 2005	23/38	Active MD^[1]^	N	NMNH	V-LD <0.3 ms	N	100% (23/23)	100% (38/38)	100% (61/61)
Don et al., 2007	23/39	Active MD^[1]^	N	NMNH	CAR≤0.95	N	100% (23/23)	75% (29/39)	84% (52/62)
De Valck et al., 2007	28/17	Definite MD, probable MD, and Possible MD	Y	NMD	V-LD <0.3 ms	N	31% (10/32)	28% (7/25)	30% (17/57)
						Y	53% (10/19)	70% (7/10)	59% (17/29)
		Definite MD				Y	100% (7/7)	70% (7/10)	82% (14/17)
Ordonez-Ordonez et al., 2009	78/32	Definite MD	N	NMD	V-LD <0.3 ms	N	31% (10/32)	100% (78/78)	80% (88/110)
						Y	37% (10/27)	100% (77/77)	84% (87/104)
		Early MD^[2]^				N	50% (1/2)	100% (78/78)	99% (79/80)
						Y	50% (1/2)	100% (77/77)	99% (78/79)
Kingma et al., 2010	22/22	Unilateral definite MD	Y	(sub-) normal contralateral ear	V-LD < 0.3 ms	N	32% (7/22)	100% (22/22)	66% (29/44)
					V-LD < 2 ms		82% (18/22)	100% (22/22)	91% (40/44)
Lee et al., 2011	47/41	Unilateral definite MD	N	NMD	V-LD < 0.3 ms	N	64% (30/47)	98% (40/41)	80% (70/88)
					CAR≤0.95		91% (43/47)	83% (34/41)	88% (77/88)
					V-LD < 0.3 ms and CAR≤0.95		62% (29/47)	98% (40/41)	78% (69/88)
					V-LD < 0.3 ms or CAR≤0.95		94% (44/47)	83% (34/41)	89% (79/88)
Shang et al., 2012	53/20	Definite MD	Y	NMNH	V-LD < 0.3 ms	N	52% (36/70)	100% (40/40)	69% (76/110)
					V-LD < 0.6 ms		100% (70/70)	93% (37/40)	97% (107/110)
					CAR≤0.95		82% (57/70)	50% (20/40)	70% (77/110)
					CAR≤0.80		60% (42/70)	97% (39/40)	74% (81/110)
Zack-Williams et al., 2012	30/0	Suspected MD	Y	NA	V-LD < 0.3 ms	N	27% (8/30)	NA	NA
					CAR≤0.95		30% (9/30)	NA	NA
Hong et al., 2013	13/15	Suspected MD^[3]^	Y	ALFHL	CAR≤0.975	N	82% (11/13)	73% (11/15)	78% (22/28)

V-LD, the cutoff of wave V latency delays in the CHAMP test; CAR, complex amplitude ratio; NMNH, non-Meniere’s disease normal hearing; NMD, non-Meniere’s disease but with other hearing or vertigo problems; MD, Meniere’s disease; ALFHL, acute low-frequency hearing loss who did not initially present with vertigo; “N/A” means data were not applicable; patients diagnosed by criteria of AAO-HNS can be divided into certain, definite, probable, and possible MD. ^[1]^The continued presence of three to four of the hallmark symptoms at the time of testing; ^[2]^possible MD evolving into a definite MD; ^[3]^ALFHL patients who experienced either a vertigo attack or a hearing fluctuation.

### 3.1 Inclusion criteria for MD patients and control subjects

Inconsistency in the diagnostic value of CHAMP may be attributed to the variability of MD cases included in the studies (20). The inclusion criteria for MD patients in previous literature are inconsistent. Some studies explicitly state that the AAO-HNS (1995) guidelines were used as inclusion criteria for MD patients (13, 15, 17–19), while others do not. In the initial study, although Don et al. (10) did not clearly state the use of the AAO-HNS guidelines for patient inclusion, they enrolled individuals who exhibited three to four hallmark symptoms at the time of testing, all of whom met the MD diagnostic criteria. This study demonstrated that the CHAMP had 100% sensitivity and 100% specificity for diagnosing MD. Don et al. (31) emphasized that this test can achieve high sensitivity and specificity when using very strict inclusion criteria for MD patients.

In the AAO-HNS (1995) guidelines, the diagnosis of MD was divided into four categories: certain MD, definite MD, probable MD, and possible MD (32). De Valck et al. (13) classified MD patients according to the AAO-HNS guidelines. When patients with definite MD, probable MD, and possible MD were included together in the MD group, the CHAMP showed lower diagnostic sensitivity (53%, excluding the non-interpretable results). Still, when only definite MD was included in the MD group, its diagnostic sensitivity was consistent with the initial study (100%). Zack-Williams et al. (18) included 30 patients, only 20 cases with vertigo and 17 cases with hearing loss, suspected to have MD without detailed classification. Therefore, the CHAMP only shows approximately 30% sensitivity. However, Hong et al. (19) included acute low-frequency hearing loss in those who did not initially present with vertigo and experienced either a vertigo attack or a hearing fluctuation during follow-up as MD group. The stricter inclusion criteria gave it an 82% sensitivity for the CHAMP. Moreover, after excluding the non-interpretable results and adjusting the abnormality criterion, the studies that included definite MD all obtained high diagnostic sensitivity for the CHAMP (from 64 to 100%) (15–17). Although the diagnosis is strictly carried out according to the guidelines, the clinical characteristics of MD are variable. Studies related to Gd-enhanced MRI of the inner ear have shown that not all MD patients have ELH (33). The above studies suggest that the diagnostic value of the CHAMP may be underestimated if patients with probable and possible MD are included (13, 18).

Meanwhile, selecting the control group may also interfere with evaluating the diagnostic value of the CHAMP (13, 14, 16). Don et al. (10) and Shang et al. (17)selected non-MD normal-hearing (NMNH) subjects in good general health and reported normal neurological status, mostly young subjects, as the control group, demonstrating ideal diagnostic specificity. The study of Don et al. (10) emphasized the diagnostic accuracy of the CHAMP as high as 100%, and Shang et al. (17) also achieved a high sensitivity of 100%, specificity of 93%, and diagnostic accuracy of 97%. However, De Valck et al. (13) and Ordóñez-Ordóñez et al. (14) included non-MD with other hearing or vertigo complaints as the control group. Even after excluding the non-interpretable CHAMP tests, there is still only 70% specificity in the study of De Valck et al. (13) and 37% sensitivity in the study of Ordóñez-Ordóñez et al. (14). Kingma et al. (15) compared the CHAMP response between the non-affected and affected ears in unilateral MD patients. They found a statistically significant difference in the latency delay between the affected ears and non-affected ears, but the diagnostic sensitivity was only 32% (7/22) (15). This may be due to the potential pathological changes in the contralateral inner ear of unilateral MD patients (34). Therefore, the choice of different control subjects can influence the evaluation of the diagnostic value of the CHAMP.

### 3.2 Indicators setting and combination

The normal range of the CHAMP variables may be different in different populations (15, 17). Adjusting the variables settings may improve the diagnostic value of the CHAMP for MD (16).

Don et al. (30) first used latency delay <0.3 ms as a criterion for CHAMP abnormalities (10) and then proposed CAR <0.95 as an additional one. Subsequent studies have found that adjusting the criteria improves the diagnostic accuracy of the CHAMP, while the appropriate criteria with the best diagnostic value vary widely according to the ROC curves of each study (15, 17). Shang et al. (17) used latency delay <0.3 ms as a criterion for CHAMP abnormalities and showed a sensitivity of only 52%. However, after resetting the criteria (latency delay <0.6 ms), the CHAMP showed 100% sensitivity, 93% specificity, and 97% diagnostic accuracy. Similarly, Kingma et al. (15) found that the sensitivity of the CHAMP improves from 32 to 82% if a criterion of <2.0 ms rather than 0.3 ms is used to determine latency delay. As for CAR, when the criterion for CAR abnormality was defined as <0.80 rather than <0.95, Shang et al. (17) achieved a specificity of 97% and a sensitivity of 60%. In the study by Hong et al. (19), in patients with acute low-frequency hearing loss not initially associated with vertigo, a CAR ≤0.975 indicated the likelihood that their clinical presentation would progress to match ELH, with a sensitivity of 82% and a specificity of 73%. These findings suggested that the normal range of CHAMP in different subjects may not be consistent due to the possible differences in the characteristics of congenital basilar membrane stiffness, TWV, and brain electrical activity (17, 35).

The impact of different parameter combinations on the diagnostic value of the CHAMP has been investigated (16). The abnormal findings in both latency delay and CAR may improve the specificity of the CHAMP in theory. However, because almost all cases with an abnormal latency delay are associated with an abnormal CAR, Lee et al. (16) obtained the same specificity as latency delay alone (98%). The abnormal findings in either latency delay or CAR can improve the sensitivity of the CHAMP. Lee et al. (16) improved the sensitivity of the CHAMP from 64 and 91 to 94% in this combination. Compared to a single parameter, combinations of multiple parameters may assist in the identification of ELH.

### 3.3 Recognition of waveforms

In the CHAMP test, wave recognition is a crucial issue, but many factors make it difficult to identify the waveforms (14, 17, 30). First, the poor synchronization of nerve fibers responsible for sensing low-frequency sounds leads to low-amplitude and broad-peak waveforms; thus, wave V cannot be identified well. Second, the identification might be interfered with by the undermasked components (10). Therefore, there may be subjective differences in waveform recognition among different studies. For that, waveform recognition of some studies was double-blinded (13, 14, 16, 20), which somewhat avoids the influence of subjective factors.

Due to the difficulty in measuring latency delay in the CHAMP, Don et al. (13) proposed the CAR in 2007. CAR represents the ratio of the amplitude of wave V in the subtracted waveform, obtained by subtracting the 0.5 kHz high-pass response from the click-alone response, compared to the amplitude in the click-alone response. Although CAR avoids the difficulty of identifying waves V under high pass noise masking, it also brought a new problem that the wave V in subtracted waveform might still be difficult to identify. Don et al. (31) pointed out that if a clear wave V could not be found in 0.5 kHz high-pass response, other high-pass responses should be used for the analysis. The results showing that the latency of wave V increases as the cochlea is progressively masked with 8, 4, 2, and 1 kHz high-pass masking noise, which are not consistent with MD, should be considered normal (non-MD) results (17, 31). This is true even when wave V in the 0.5 kHz high-pass response cannot be identified and the latency delay cannot be calculated.

The difficulty in waveform recognition not only affects the evaluation of CHAMP diagnostic value but also impacts its application. In De Valck et al.’s study, half of the waveforms are difficult to interpret. Their results provide a sensitivity of 31.3% and a specificity of 28%, including the non-interpretable CHAMP data (13). If the non-interpretable CHAMP responses are excluded, the results were 53 and 70%, which is still below practical use (13). However, directly excluding these waveforms also carries the risk of increasing bias.

### 3.4 Impact of SNHL on the CHAMP

The CHAMP variables are based on differences in ABR waveforms under different high-pass noise masking conditions. SNHL occurs in patients with non-MD and can also cause abnormalities in ABR, which affects CHAMP results.

The stimulus of CHAMP is 60 dB nHL click (10), and the ABR masked by high-pass noise is completely induced by the low-frequency region of the click. Therefore, the CHAMP is unsuitable for patients with hearing thresholds exceeding 50–55 dB, low-frequency hearing loss, conductive or mixed hearing loss, and hyperacusis (16). Kim et al. (20) examined the influence of factors such as SNHL on the CHAMP and found that non-MD subjects with SNHL also showed a significantly shorter latency delay, similar to MD patients.

Moreover, Kim et al. (20) proposed that the high sensitivity and specificity may be partly the result of hearing loss in MD patients, rather than ELH itself. Therefore, when using CHAMP to assist in the diagnosis of MD, SNHL needs to be considered. Developing personalized criteria based on the degree of SNHL in the subjects may improve the diagnostic accuracy of CHAMP. More reasonable and larger sample size studies need to be designed to further clarify the impact of SNHL and ELH on CHAMP results.

### 3.5 Associations of CHAMP and ECochG

Clinically, many neurotological evaluations, including the ECochG and glycerol tests, have been used to detect the presence of ELH in MD patients. Therefore, a few studies have compared the clinical values of the CHAMP and ECochG tests in diagnosing MD (16, 18). Zack-Williams et al. (18) found that the ECochG test had a higher sensitivity, ranging from 43% (SP/AP > 0.4) to 63% (SP/AP > 0.35), compared to the CHAMP (approximately 30%) in diagnosing unilateral definite MD. However, Lee et al. (16) found that ECochG had a sensitivity of only 21%, a specificity of 97%, and a diagnostic accuracy of 62%. The sensitivity, specificity, and diagnostic accuracy of the CHAMP were 64, 98, and 80%, respectively, for latency delay and 91, 83, and 88%, respectively, for the CAR. They found that the sensitivity and diagnostic accuracy of the CHAMP were significantly higher than the corresponding values for ECochG (16). Many factors may contribute to the different sensitivity of ECochG, including electrode configurations, recording sites, stimulus choices, and recording protocols. In addition, Zack-Williams et al. (18) explained that MD pathology may be responsible for the different sensitivities of the ECochG and CHAMP in the studies mentioned above. However, both ECochG and CHAMP are auditory electrophysiological examinations, and their results are influenced by stimulation parameters, recording methods, and hearing levels. Further research with larger sample sizes is needed to verify the value of ECochG and CHAMP in MD diagnosis.

## 4 Current status and prospects of the CHAMP

Determining ELH is crucial for diagnosing MD. Currently, there are two approaches: audio-vestibular and imaging evaluations. Compared to ECochG and glycerol tests, the CHAMP is a relatively new electrophysiologic technique. Unlike the glycerol test, the CHAMP does not cause discomfort from oral glycerol administration, and the procedure is relatively simple compared to the ECochG. Furthermore, in the last 10 or more years, imaging has been an important technique worldwide for determining ELH in diagnosing MD. In contrast to electrophysiologic evaluation, imaging techniques can observe the patient’s ELH *in vivo*. ELH demonstrated by Gd-enhanced MRI of the inner ear is not only observed in definite MD but is also common in other hydropic ear diseases such as probable MD, low-frequency SNHL, and acoustic neuroma (36). In contrast, this review shows that the ability of the CHAMP to recognize ELH has only been well validated in definite MD, but its potential to reflect more pathological and physiological states of the inner ear may need further exploration. Therefore, such differences in morphological and functional indicators of ELH for MD patients warrant further investigation.

Several limitations exist in the current research on the CHAMP for diagnosing MD. First, the published literature on the CHAMP to date is not abundant, and it is mainly focused on the years from 2005 to 2015. The smaller number of literature may not be beneficial for drawing more unbiased conclusions about the clinical value of CHAMP in diagnosing MD. Moreover, the CHAMP has been rarely documented in other hydropic ear diseases. Second, in recent years, with the extensive use of MRI inner ear imaging, the application of audiological techniques to evaluate ELH has become unfashionable, which is one of the reasons why CHAMP has been less frequently described. Furthermore, unfortunately, there are no studies that have explored the diagnostic significance of CHAMP in MD in the context of Gd-enhanced MRI of the inner ear. To improve the diagnostic accuracy of definite or even probable or possible MD, further integration of imaging and audiological methods may be useful in future research.

Moreover, at present, there has been no attempt to adjust the stimulation strategy, which could further optimize CHAMP and enhance its diagnostic value. Developing better stimulus strategies may become a further research direction in the future.

It is worth noting that the CHAMP does not directly reflect ELH, and the traveling wave velocity and response characteristics of the basement membrane directly influence its results. Therefore, its role in reflecting the pathological and physiological changes of the basement membrane rather than ELH itself appears more promising. Interestingly enough, the potential of CHAMP can be well validated in animal models, although there are currently no published animal studies related to CHAMP, which may be highly worthy of further research for inner ear disorders.

## 5 Conclusion

Previous studies have shown considerable variability in the diagnostic value of the CHAMP for MD. We highlight the heterogeneity of these studies, such as subject inclusion criteria, variable settings, and waveform identification, which may account for the significant differences in these diagnostic values. Currently, the CHAMP is not a popular diagnostic approach for MD, but it may still be useful for examining the pathophysiology underlying hydropic ear diseases as the procedure can assess the TWV of the basilar membrane.
